# Predicting Adverse Events in Blunt Chest Trauma: A Novel Nomogram Integrating Vitals, Hemogram, and Comorbidities

**DOI:** 10.1002/kjm2.70088

**Published:** 2025-08-21

**Authors:** Chang‐Lun Huang, Hui‐Min Hsieh, Chew‐Teng Kor, Ming‐Chung Chou, Po‐Chih Chang, Ting‐Wei Chang, Chao‐Wen Chen

**Affiliations:** ^1^ Division of General Thoracic Surgery, Department of Surgery Changhua Christian Hospital Changhua Taiwan; ^2^ Graduate Institute of Biomedical Sciences China Medical University Taichung Taiwan; ^3^ Department of Public Health Kaohsiung Medical University Kaohsiung Taiwan; ^4^ Center for Big Data Research Kaohsiung Medical University Kaohsiung Taiwan; ^5^ Big Data Center, Changhua Christian Hospital Changhua Taiwan; ^6^ Graduate Institute of Statistics and Information Science National Changhua University of Education Changhua Taiwan; ^7^ Department of Medical Imaging and Radiological Sciences Kaohsiung Medical University Kaohsiung Taiwan; ^8^ Department of Medical Research Kaohsiung Medical University Hospital Kaohsiung Taiwan; ^9^ School of Medicine, College of Medicine, National Sun Yat‐Sen University Kaohsiung Taiwan; ^10^ Division of Thoracic Surgery, Department of Surgery Kaohsiung Medical University Hospital/Kaohsiung Medical University Kaohsiung Taiwan; ^11^ Weight Management Center Kaohsiung Medical University Hospital/Kaohsiung Medical University Kaohsiung Taiwan; ^12^ Department of Sports Medicine, College of Medicine Kaohsiung Medical University Kaohsiung Taiwan; ^13^ Ph. D. Program in Biomedical Engineering, College of Medicine Kaohsiung Medical University Kaohsiung Taiwan; ^14^ Faculty of Medicine, College of Medicine Kaohsiung Medical University Kaohsiung Taiwan; ^15^ Division of Trauma and Surgical Critical Care, Department of Surgery Kaohsiung Medical University Hospital/Kaohsiung Medical University Kaohsiung Taiwan

**Keywords:** adverse event, blunt chest trauma, emergency department, nomogram, prediction

## Abstract

Blunt chest trauma (BCT) is common and frequently associated with adverse complications. Beyond merely impeding regular respiration, adverse events (AEs) such as hemothorax or pneumothorax can hinder the patient's recovery. Herein, we aim to validate potential predictive factors for AEs among adults with BCT who were admitted concurrently through the dataset focusing on the limited information available upon their arrival at the emergency department (ED). Seventeen variables—including patients' demographics, comorbidities, and vital signs/hemogram data upon arrival at the ED—were investigated. A penalized logistic regression model was applied to the derivation cohort and validated in a subgroup using the same dataset (80%:20%). In addition, we employed the least absolute shrinkage and selection operator (LASSO) logistic regression to develop a nomogram, which enhances the accuracy of estimating individual probabilities for AEs after admission for BCT. Our retrospective review encompassed 3,668 adult patients between 2017 and 2021, and the incidence of AEs was 15.6% (572 out of 3,668). Penalized logistic regression was conducted both without and with the hemogram data (Model 1 and Model 2), yielding relatively satisfactory results (*R* ^2^: 0.271 vs. 0.291; area under the curve: 0.784 vs. 0.797, respectively). Despite the model's relatively high predictive value in the derivation cohort, the validation data still maintained an acceptable accuracy of 0.7456 and 0.7049, respectively. Employing our penalized logistic regression analysis, the recently formulated nomogram exhibited proficiency in predicting AEs following BCT. This effectiveness was achieved by integrating vital signs, hemogram data, and comorbidities recorded upon their arrival at the ED.

## 1 Introduction

Trauma stands as the foremost cause of mortality and disability among young adults, consistently ranking among the top six common causes of death in Taiwan for more than a decade, accounting for approximately 30 deaths per 100,000 persons annually [1, 2]. Of these cases, two‐thirds involved chest trauma, ranging from simple rib fractures to penetrating injuries affecting the heart or tracheobronchial structures. Blunt chest trauma (BCT), which accounts for 90% of incidents, manifests with varying severity; however, only a minority—less than 10%—necessitate surgical intervention. Following a head injury, chest trauma has the second‐highest mortality rate, underscoring the critical importance of immediate and effective management to prevent avoidable deaths. Polytraumatized patients commonly present with chest trauma, constituting 60% of cases and carrying a mortality rate of up to 25% [3, 4]. The early identification of patients with poor prognostic indicators and timely intervention is crucial for improving outcomes in this population.

Several risk factors for unfavorable outcomes and mortality have been suggested in patients with BCT, such as senescence, radiologically determined lung injuries, associated extra‐thoracic injuries, or multiple rib fractures, and these contribute to an improved level of care [5, 6, 7]. Considering the multifactorial nature of BCT, Battle et al. developed and validated an efficient prognostic risk score to predict complications and guide subsequent management [6]. This score is based on factors such as age, the number of rib fractures, the presence of chronic lung disease, pre‐injury use of anticoagulants, and oxygen saturation levels [8]. A meta‐analysis updated in 2023 also confirmed similar risk factors for mortality following BCT. Additional novel risk factors, which were not previously reported, included an increased Injury Severity Score, the need for mechanical ventilation, extremes of body mass index, and smoking status [9]. A series of scoring systems based on radiography were also proposed to forecast outcomes for individuals having BCT [5, 10, 11]. Biomarkers and cytokines such as interleukin (IL) 1β, IL6, IL8, IL10, and tumor necrotic factor‐α (TNF‐α) were also explored as predictive tools [12]. However, despite these advancements, a clinically applicable tool for emergency medicine clinicians, especially the application of on‐arrival data to predict adverse events or complications, is currently unavailable [13]. In this context, hemogram results were included in our model as part of “on‐arrival data” because they are routinely collected and promptly made available in most emergency settings, in contrast to sonographic findings that could be operator dependent and not consistently performed across cases [14, 15].

Nomograms, as graphical calculation instruments, offer a unique and versatile approach based on various functions, including logistic regression or Cox hazard ratio regression models [16, 17]. Accommodating both continuous and categorical variables, nomograms visually represent the impact of variables on specific outcomes, assigning risk points according to their prognostic or predictive significance. This flexibility distinguishes nomograms from look‐up tables or decision trees, making them particularly suitable for processing continuously coded variables and enabling comprehensive data stratification. By not just extracting maximal information from data, nomograms could provide accurate predictions and offer the added advantage of visualizing predictive factors and their contributions [18], making them a valuable tool in clinical scenarios and aiding communication with family members and patients. To this end, the recent increase in the use of nomograms to forecast the prognosis of patients experiencing BCT has shown relatively favorable results [19, 20].

This study aimed to validate the potential predictive factors of adverse events (AEs) for adult patients experiencing BCT with concurrent admission upon arrival at the emergency department (ED) by integrating vital signs, hemogram data, and existing comorbidities, utilizing data from a tertiary medical center in Taiwan [13, 21, 22].

## 2 Methods

This retrospective cohort study was conducted at a tertiary medical center in Taiwan. Approval for the study was obtained from the Institutional Review Board of the hospital (IRB No. 230304). Given the retrospective nature of the study, the requirement for written informed consent was waived by the Ethics Committee. All the methods were conducted following the principles outlined in the Declaration of Helsinki and the relevant guidelines and regulations. The electronic medical records of patients with BCT between January 2017 and December 2021 were systematically reviewed and analyzed.

In this study, we included adult patients aged over 18 years who presented with BCT to the ED and excluded patients who experienced mortality in the ED, those undergoing surgery/management for other injuries, those transferring to other facilities without admission, and individuals with incomplete essential records. In this context, post‐BCT AEs were defined as the occurrence of any of the following: pneumonia, acute respiratory failure, tracheostomy, intensive care unit (ICU) stay, or in‐hospital death. To mitigate confounding from extra‐thoracic injuries, we included only patients with isolated or predominant BCT and excluded those with severe concomitant head injuries or other major trauma requiring immediate surgical intervention.

### 2.1 Data Collection

There were 17 variables in the comprehensive analysis, encompassing patient characteristics such as sex and age, the Charlson Comorbidity Index, the presence of comorbidities (chronic obstructive pulmonary disease, diabetes, chronic kidney disease, or cerebrovascular accident), vital signs upon arrival at the ED (systolic blood pressure, diastolic blood pressure, pulse rate, body temperature, respiratory rate, oxygen saturation), and hemogram parameters (the white blood cell count, hemoglobin level, hematocrit, platelet count) [13, 21, 22]. In our study design, hemogram parameters were classified as “on‐arrival data” because blood samples were systematically drawn at triage and processed promptly as part of routine ED protocols. These values were recorded before the commencement of any major resuscitative measures, thereby capturing the patient's physiological baseline at the time of ED presentation. This classification is consistent with the standard clinical workflows observed across the participating Level I trauma centers. During the study period, 19,183 patients with BCT were admitted, and 3,668 of them were ultimately included.

### 2.2 Statistical Analysis

Mean values with standard deviations (for normally distributed data) or median values with interquartile ranges (for non‐normally distributed data) were utilized to present continuous data, and comparisons of such data were conducted using Student's *t*‐test or the Mann–Whitney U test, respectively. Qualitative data were expressed as frequencies (percentages) and compared using the Chi‐square test or Fisher's exact test. Microsoft Excel 2016 (Microsoft Corp., Wash, USA) was employed for data collection and table formation.

To construct the predictive model for AEs following BCT, we employed the least absolute shrinkage and selection operator regression (LASSO) to select variables [23, 24]. The optimal penalization factor was identified through the “pentrace” function within Harrell's R package “rms” to prevent overfitting. Internal model validation employed the bootstrapping approach, chosen for its efficiency over the split‐data and cross‐validation methodologies [25]. Bootstrapping mimics the procedure of generating samples from an underlying population by randomly selecting samples with replacement from the original dataset. Constructing and validating the model can be done using 100% of the subjects through the bootstrapping method.

However, employing cross‐validation or data splitting involves utilizing a subset of subjects for model construction and the remainder for validation. This approach can lead to unstable and biased performance estimates. A nomogram was utilized to present the risk‐predictive model for AEs for BCT patients during their admission. The assessment of model performance involved two main aspects: (1) Discriminatory capacity was gauged using the area under the receiver operating characteristic (ROC) curve (AUC), and bias‐corrected AUC was computed through 1000 bootstrap iterations. (2) Calibration ability was analyzed using the calibration plot and the Brier score. Furthermore, an optimal cut‐off threshold was identified to evaluate the positive predictive value and negative predictive value for assessing clinical utility. A sensitivity analysis was performed on the entire cohort.

Two‐tailed tests were conducted, and *p*‐values of < 0.05 were considered statistically significant. The ROC curve was utilized to determine the optimal cut‐off value. Statistical analyses were performed using R (version 4.1.0 accessed on 18 May 2021; The Comprehensive R Archive Network: http://cran.r‐project.org).

## 3 Results

In this retrospective study involving 3,668 eligible patients, the incidence of AEs was 15.6% (572/3,668). Of these, 2,245 (61.2%) were male, and individuals who experienced AEs following BCT were older than those without AEs (62 ± 19 vs. 58 ± 18 years; *p* < 0.001). The occurrence of comorbidities did not differ significantly between individuals with and without AEs, except for a higher percentage of patients with chronic kidney disease in the group with AEs (13% vs. 9%; *p* = 0.010). Upon arrival, vital signs and hemogram data indicated comparatively lower blood pressure, elevated pulse rate, decreased body temperature, lower blood oxygen saturation levels, higher white blood cell count, reduced hemoglobin, and a lower platelet count in the group experiencing AEs (Table 1).

**TABLE 1:** Demographic and characteristics variables for patients with blunt chest trauma.

	No adverse event (*n* = 3096)	One or more adverse events (*n* = 572)	*p*	Derivation cohort (*n* = 2,933)	Validation cohort (*n* = 735)	*p*
Age (years)	58 ± 18	62 ± 19	< 0.001	58 ± 19	60 ± 18	0.046
Sex (male)	1,844 (60%)	401 (70%)	< 0.001	1,799 (61%)	446 (61%)	0.224
Charlson comorbidity index	1.5 ± 2.4	1.7 ± 2.7	0.022			
Category						
0	1679 (54%)	310 (54%)	0.022	1,581 (54%)	408 (56%)	0.569
1–2	731 (24%)	115 (20%)		682 (23%)	164 (22%)	
≥ 3	686 (22%)	147 (26%)		670 (23%)	163 (22%)	
COPD	377 (12%)	91 (16%)	0.017	377 (13%)	91 (12%)	0.221
DM	523 (17%)	100 (17%)	0.776	511 (17%)	112 (15%)	0.825
CKD	294 (9%)	75 (13%)	0.010	294 (10%)	75 (10%)	0.062
CVA	363 (12%)	65 (11%)	0.860	339 (12%)	89 (12%)	0.275
SYS (mmHg)	132 ± 15	126 ± 24	< 0.001	131 ± 17	131 ± 19	0.731
DIA (mmHg)	76 ± 10	68 ± 14	< 0.001	74 ± 11	74 ± 12	0.975
PULSE (per minute)	80 ± 12	90 ± 18	< 0.001	82 ± 14	82 ± 14	0.627
TEMP (°C)	37 ± 0.4	36 ± 1.1	< 0.001	37 ± 0.59	37 ± 0.6	0.374
RR (per minute)	18 ± 1.8	18 ± 3.5	< 0.001	18 ± 2.2	18 ± 2	0.681
SpO2 (%)	97.1 ± 1.5	96.6 ± 4.3	< 0.001	97.1 ± 2.2	96.9 ± 2.1	0.012
WBC (10^3^/μL)	8.8 ± 4.2	11 ± 5.5	< 0.001	9.5 ± 4.7	9.8 ± 4.8	0.150
Hgb (g/dL)	12 ± 2.2	11 ± 2.5	< 0.001	12 ± 2.4	12 ± 2.2	0.922
HCT (%)	36 ± 6.4	32 ± 7.3	< 0.001	35 ± 7	35 ± 6.6	0.817
Platelet (10^3^/μL)	197 ± 96	142 ± 79	< 0.001	181 ± 91	188 ± 108	0.165
Adverse events						
Pneumonia	—	132 (23%)	—	104 (4%)	28 (4%)	0.184
ARF	—	247 (43%)	—	193 (7%)	54 (7%)	0.490
Tracheostomy	—	56 (10%)	—	48 (2%)	8 (1%)	0.640
ICU stay	—	204 (36%)	—	160 (5%)	44 (6%)	0.363
In‐hospital death	—	263 (46%)	—	206 (7%)	57 (8%)	0.456

*Note*: Adverse events: the one of the events of pneumonia, ARF, tracheostomy ICU stay, and in‐hospital death.

Abbreviations: ARF: acute respiratory failure; CKD: chronic kidney disease; COPD: chronic obstructive pulmonary disease; CVA: cerebrovascular accident; DIA: diastolic blood pressure; DM: diabetes mellitus; HCT: hematocrit; Hgb: hemoglobin; ICU: intensive care unit; PULSE: pulse rate; RR: respiratory rate; SpO2: blood oxygen saturation levels; SYS: systolic blood pressure; TEMP: body temperature; WBC: white blood cell count.

The penalized logistic regression model for anticipating AEs in the derivation cohort was partitioned into two groups: one without the hemogram (Model 1) and the other with the hemogram (Model 2). These models exhibited *R* ^2^ values of 0.271 versus 0.291, AUC values of 0.784 versus 0.797, and Brier scores of 0.103 versus 0.150, respectively (Table 2).

**TABLE 2:** Logistic regression model for predicting adverse events in the derivation cohort.

	Model 1: penalized logistic model without hemogram (penalization factor = 8.00)			Model 2: penalized logistic model with hemogram (penalization factor = 9.90)		
	Coefficient	OR (95% CI)	*p*	Coefficient	OR (95% CI)	*p*
Intercept	20.142			39.687		
Age	0.015	1.02 (1.01, 1.02)	< 0.001	0.002	1.00 (0.99, 1.01)	0.633
Male	0.576	1.78 (1.39, 2.27)	< 0.001	0.445	1.56 (1.18, 2.06)	0.002
Comorbidities						
COPD	0.354	1.42 (1, 2.02)	0.048	0.216	1.24 (0.83, 1.85)	0.286
CVA	−0.156	0.86 (0.58, 1.25)	0.422	0.116	1.12 (0.77, 1.65)	0.553
DM	−0.108	0.9 (0.63, 1.28)	0.553	0.22	1.25 (0.84, 1.84)	0.270
CKD	0.36	1.43 (0.95, 2.15)	0.084	−0.149	0.86 (0.55, 1.34)	0.508
Charlson comorbidity index						
0	0	1		0	1	
1–2	−0.049	0.95 (0.69, 1.31)	0.763	−0.321	0.73 (0.50, 1.04)	0.083
≥ 3	−0.227	0.8 (0.51, 1.25)	0.326	−0.385	0.68 (0.43, 1.08)	0.103
Vital signs upon arrival						
SYS	0.013	1.01 (1, 1.02)	0.007	−0.002	1.00 (0.99, 1.01)	0.708
DIA	−0.072	0.93 (0.92, 0.94)	< 0.001	−0.019	0.98 (0.97, 1.00)	0.023
PULSE	0.058	1.06 (1.05, 1.07)	< 0.001	0.015	1.02 (1.00, 1.03)	0.005
TEMP	−0.542	0.58 (0.47, 0.71)	< 0.001	−0.371	0.69 (0.51, 0.94)	0.018
RR	−0.149	0.86 (0.82, 0.91)	< 0.001	−0.060	0.94 (0.89, 1.00)	0.057
SPO_2_	−0.02	0.98 (0.93, 1.03)	0.445	−0.241	0.79 (0.71, 0.87)	< 0.001
Hemogram upon arrival						
WBC				0.125	1.13 (1.1, 1.17)	< 0.001
Hgb				−0.279	0.76 (0.52, 1.09)	0.136
HCT				0.015	1.01 (0.9, 1.15)	0.816
Platelet				−0.007	0.993 (0.991, 0.995)	< 0.001
Brier	0.103			0.150		
*R* ^2^	0.271			0.291		
AUC	0.784			0.797		
Bias‐corrected AUC	0.777			0.786		
Predicted score	−2.19 (−2.81, −1.47)			−1.35 (−2.16, −0.48)		
Predicted probability	0.10 (0.06, 0.19)			0.21 (0.10, 0.38)		

Abbreviations: ARF, acute respiratory failure; AUC, Area under curve; CKD, chronic kidney disease; COPD, chronic obstructive pulmonary disease; CVA, cerebrovascular accident; DIA, diastolic blood pressure; DM, diabetes mellitus; HCT, hematocrit; Hgb, hemoglobin; ICU, intensive care unit; PULSE, pulse rate; RR, respiratory rate; SpO2, blood oxygen saturation levels; SYS, systolic blood pressure; TEMP, body temperature; WBC, white blood cell count.

**FIGURE 1:**
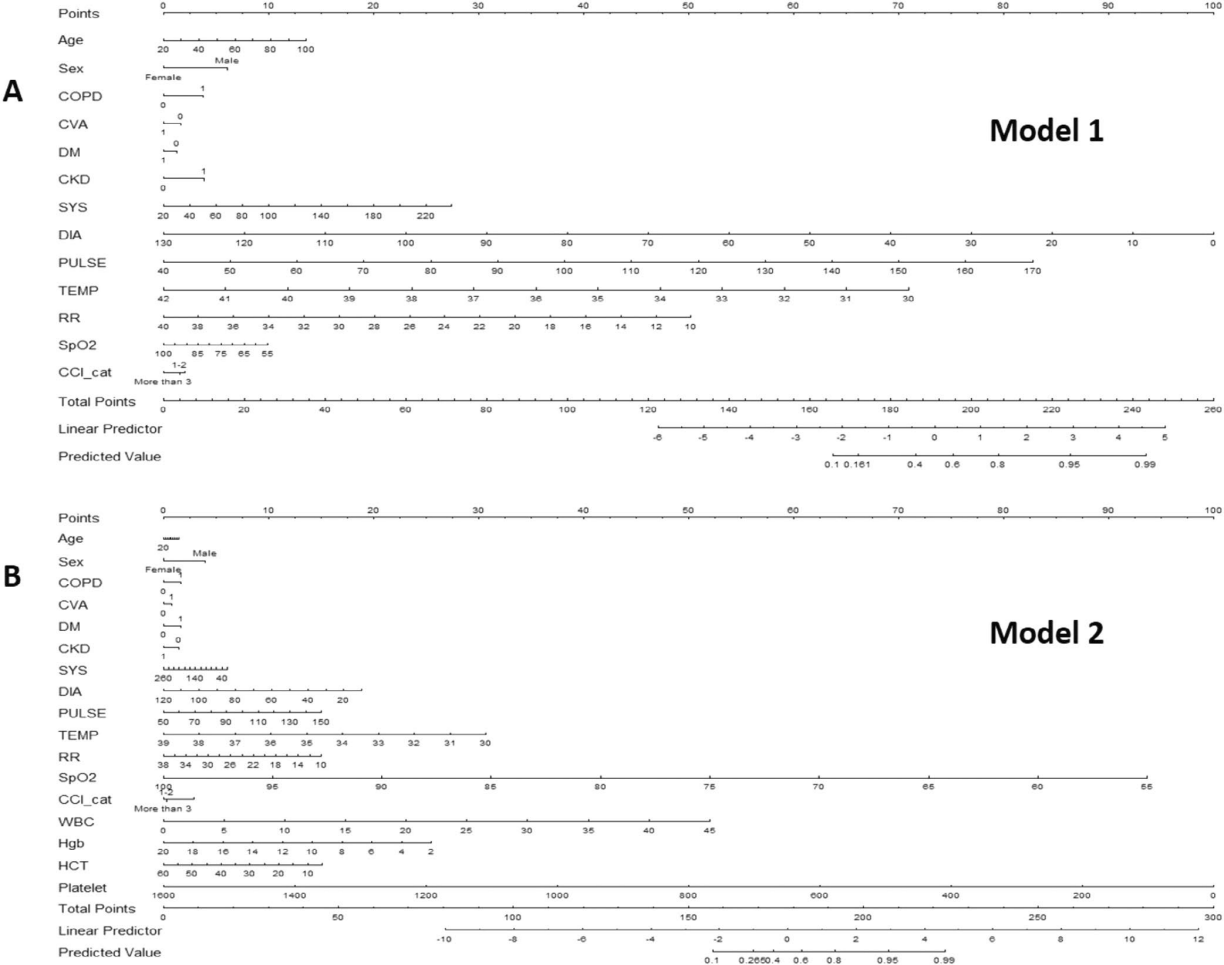
Nomograms for predicting adverse events after blunt chest trauma (A) Model 1 (without hemogram) (B) Model 2 (with hemogram). CCI cat: Charlson Comorbidity Index category; CKD: Chronic kidney disease; COPD: Chronic obstructive pulmonary disease; CVA: Cerebrovascular accident; DIA: Diastolic blood pressure; DM: Diabetes mellitus; HCT: Hematocrit; Hgb: Hemoglobin; PULSE: Pulse rate; RR: Respiratory rate; SpO2: Blood oxygen saturation levels; SYS: Systolic blood pressure; TEMP: Body temperature; WBC: White blood cell count.

Moreover, the two comprehensive nomograms—Model 1 and Model 2—aim to predict AEs following BCT (Figure 1). Model 1, which excludes the hemogram data, incorporates essential variables such as age, sex, the Charlson Comorbidity Index, heart rate, blood pressure, and respiratory rate. Each variable aligns with a specific score on the nomogram, cumulatively leading to a total score indicative of the patient's risk probability on a calibrated scale (Figure 1A). Conversely, Model 2 extends the predictive capability by including hemogram parameters and offers a more nuanced risk assessment (Figure 1B).

**FIGURE 2:**
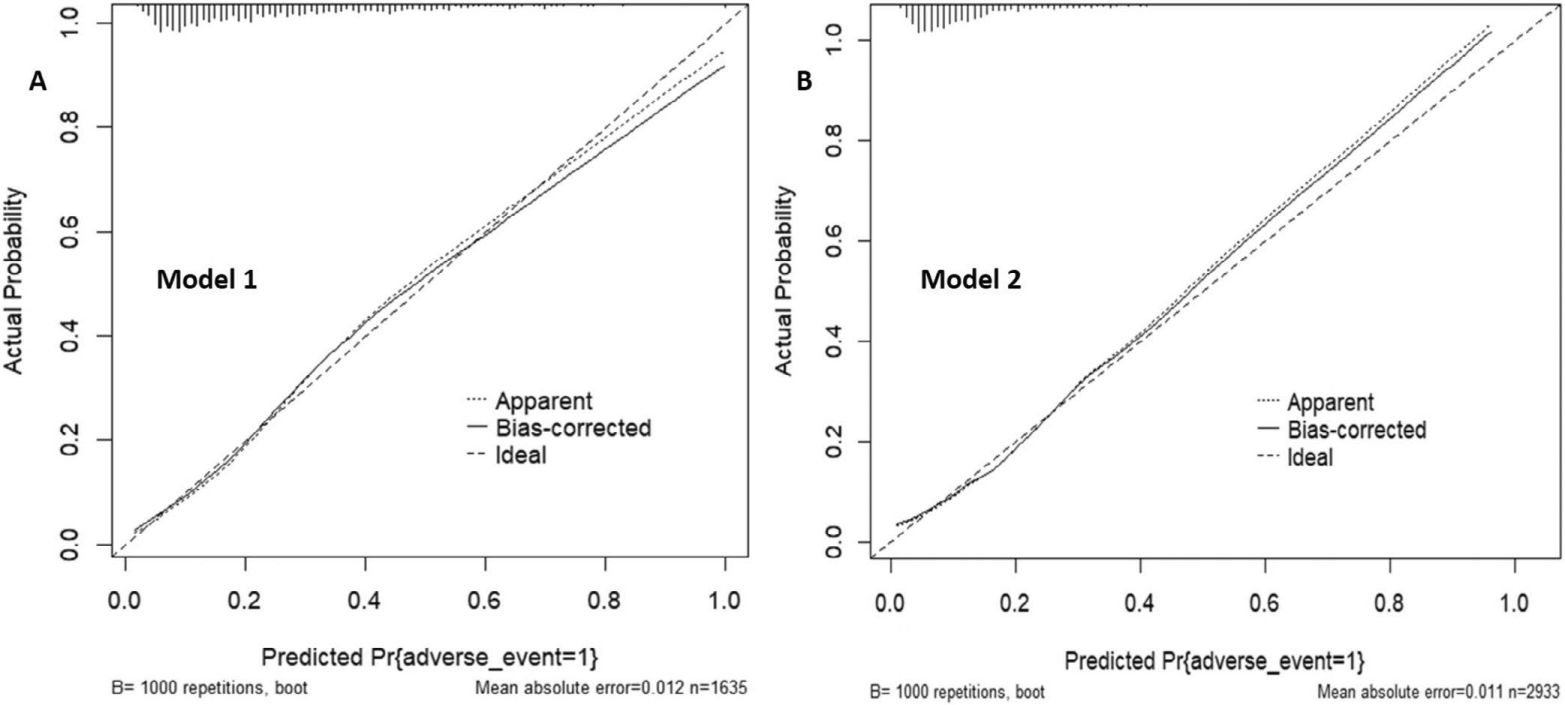
Internal calibration plot for the predictive nomogram by 1000 bootstrapping in the derivation cohort.

Model 1 and Model 2 exhibited Brier scores of 0.103 and 0.150, respectively (Table 2). The Hosmer–Lemeshow goodness‐of‐fit tests in the derivation cohort, with chi‐square values of 9.70 (Model 1) and 6.86 (Model 2) with *p* = 0.286 for Model 1 and *p* = 0.552 for Model 2, indicate a good fit for both models (Figure S1). To further assess any deviation between the observed and predicted events, we internally validated the models through bootstrapping (1000 iterations) of the calibration plot, slope, intercept, and AUC. The original AUCs were 0.7844 and 0.7967, while the bias‐corrected estimates were 0.7760 and 0.7858 for Model 1 and Model 2, respectively (Table 3). To enhance the model fit and generate a new internal calibration plot through 1000 bootstrap resamples within the derivation cohort, a penalty was introduced using the “pentrace” function in Harrell's R package “rms” [25]. The mean absolute error was 0.012 for Model 1 and 0.011 for Model 2 (Figure 2).

**TABLE 3:** Accuracy of the prediction models (Model 1: penalized logistic model without hemogram; Model 2: penalized logistic model with hemogram).

Model	Cutoff point at predicted probability	AUC	Sensitivity	Specificity	PPV	NPV	F1 score	ACC
Derivation cohort								
Model 1	0.1607	0.7844	0.6718	0.7779	0.3582	0.9277	0.4673	0.7613
Model 2	0.2645	0.7967	0.7506	0.7255	0.4962	0.8898	0.5974	0.7321
Validation cohort								
Model 1	0.1607	0.7760	0.6696	0.7597	0.3407	0.9253	0.4516	0.7456
Model 2	0.2645	0.7858	0.7156	0.7010	0.4643	0.8719	0.5632	0.7049

Abbreviations: ACC: accuracy; AUC: area under curve; NPV: negative predictive value; PPV: positive predictive value.

## 4 Discussion

Over a 5‐year period at a single Level I trauma center in Taiwan, this retrospective cohort study stands as a unique initiative in constructing a prediction model for AEs following BCT (3,668 patients), based on vitals and hemogram data collected upon arrival to the ED. Various factors, including age, sex, Charlson Comorbidity Index, presence of comorbidities, vital signs upon admission, and hemogram parameters, emerged as significant predictive factors in our analysis. Employing penalized logistic regression, we developed a nomogram to predict AEs, providing clinicians with a valuable and reasonably accurate resource.

Basically, the inclusion of hemogram data as part of the predictive model was based on its availability and clinical utility in emergency settings. Hemogram values were systematically drawn at triage and processed without delay as part of routine ED protocols [14]. These values were recorded before the commencement of any major resuscitative measures, thereby capturing the patient's physiological baseline at the time of ED presentation. Although we acknowledge that certain hemogram parameters may be influenced by both injury severity and early resuscitative efforts, this classification is consistent with the standard clinical workflows observed across the participating Level I trauma center and supports the use of hemogram parameters as valid “on‐arrival” data.

BCT is frequently associated with AEs—such as pneumonia, respiratory failure, and even death—and there is limited evidence‐based guidelines available to aid in the management, investigate reasons for readmission, or determine disposition for individuals with non‐severe, non‐immediately life‐threatening injuries at ED [3, 4, 26, 27, 28]. In light of the clinical importance of nonmajor BCT and its associated care, various scoring systems have been proposed to aid in forecasting complications and directing the management thereof. In addition to incorporating variables such as age, oxygen saturation levels, and pre‐existing comorbidities, the inclusion of rib fractures with specific patterns (number/laterality/displacement) or associated pulmonary contusion was also considered for additional validation [5, 8, 10, 11]. However, it is important to note that all these predictive risk scores were developed using correlated radiological evidence, ensuring an accurate representation of BCT severity.

Recently, pathway‐driven care using risk models for BCT has gained popularity [5, 8, 10, 11]. Considering the predominant risk factors of the elderly or the presence of pre‐existing conditions for AEs, ED clinicians, who are responsible for ensuring safe clinical decision‐making for BCT patients, may face difficulties deciding between direct discharge or selecting an appropriate disposition, even in the absence of radiology documentation [9, 22, 29]. Therefore, it is crucial to have an effective approach—even a trigger system—for ED clinicians to recognize potentially deteriorating patients following BCT using only readily available triage data, including patient demographics, past medical history, and vital signs [13]. To achieve this objective, we utilized a penalized logistic regression model to establish cohorts, validated the model, and employed LASSO logistic regression to construct a nomogram. This nomogram was developed by integrating vital signs at triage, hemogram parameters, and comorbidities, yielding satisfactory results. The study involved a population of 3,668 patients, and the incidence of post‐BCT AEs was 15.6%.

Here, we conducted penalized logistic regression models, both with and without hemogram variables, with the goal of predicting AEs in BCT patients, emphasizing its clinical significance [21]. Our findings revealed notable distinctions between Model 1, which excludes hemogram data, and Model 2, which incorporates these crucial variables. First, the performance metrics—including the AUC—underscore a slight advantage of Model 2 over Model 1 in both the derivation cohort (0.7967 vs. 0.7844) and the validation cohort (0.7858 vs. 0.7760). This suggests that the inclusion of hemogram data enhances the model's discriminatory power. Moreover, the optimal cut‐off point for the predicted probabilities in Model 2 (0.2645) outperformed that of Model 1 (0.1607), demonstrating a balanced compromise between sensitivity and specificity (Table 3). The utilization of a higher penalization factor in Model 2 (9.90) also appears to contribute to improved model performance while maintaining model parsimony. Our results highlight the critical role of hemogram integration and proper threshold determination in refining the predictive accuracy of our models. This offers valuable insights for clinicians and researchers, emphasizing the potential benefits of incorporating comprehensive patient information to enhance risk stratification in BCT patients. Further research could explore additional variables and refine the models for even greater clinical applicability.

Nevertheless, this study has some limitations that should be mentioned. First, this was a retrospective study conducted at a single tertiary medical center, and the reliance on electronic medical records may have introduced selection bias. Second, no injury severity score or abbreviated injury scale was included, and the unavailability of radiological documentation may hinder the comprehensive interpretation of the nomogram. Finally, we did not perform external validation. Although our nomogram demonstrated strong internal validity, it should be interpreted cautiously in broader clinical settings. Therefore, we recognize the need for external validation and prospective evaluation. In the future, we will conduct a larger, prospective, multicenter study to assess the real‐world applicability and generalizability of this nomogram across diverse trauma care environments.

## 5 Conclusion

Using our penalized logistic regression analysis, the newly developed nomogram demonstrated effective performance in forecasting AEs in patients with BCT. This was achieved by incorporating vital signs, hemogram data (as obtained immediately upon ED arrival), and pre‐existing comorbidities. The model has the potential to assist clinicians in early risk stratification and therapeutic decision making. Future prospective studies are warranted to validate this tool in clinical practice.

## Conflicts of Interest

The authors declare no conflicts of interest.

## Supporting information


**Figure S1:** The distribution of the predictor values by adverse events and receiver operating characteristic (ROC) curve for predicting adverse events after blunt chest trauma (A) Model 1: penalized logistic model without hemogram (penalty factor = 8.00) (B) Model 2: penalized logistic model with hemogram (penalty factor = 9.00).

## Data Availability

The data that support the findings of this study are available on request from the corresponding author. The data are not publicly available due to privacy or ethical restrictions.
