# Supplementary figures and images for: Predicting Adverse Events in Blunt Chest Trauma: A Novel Nomogram Integrating Vitals, Hemogram, and Comorbidities
Source: Kaohsiung J Med Sci. 2025 Aug 21;41(12):e70088. doi: 10.1002/kjm2.70088 (PMC12694571; doi:10.1002/kjm2.70088)

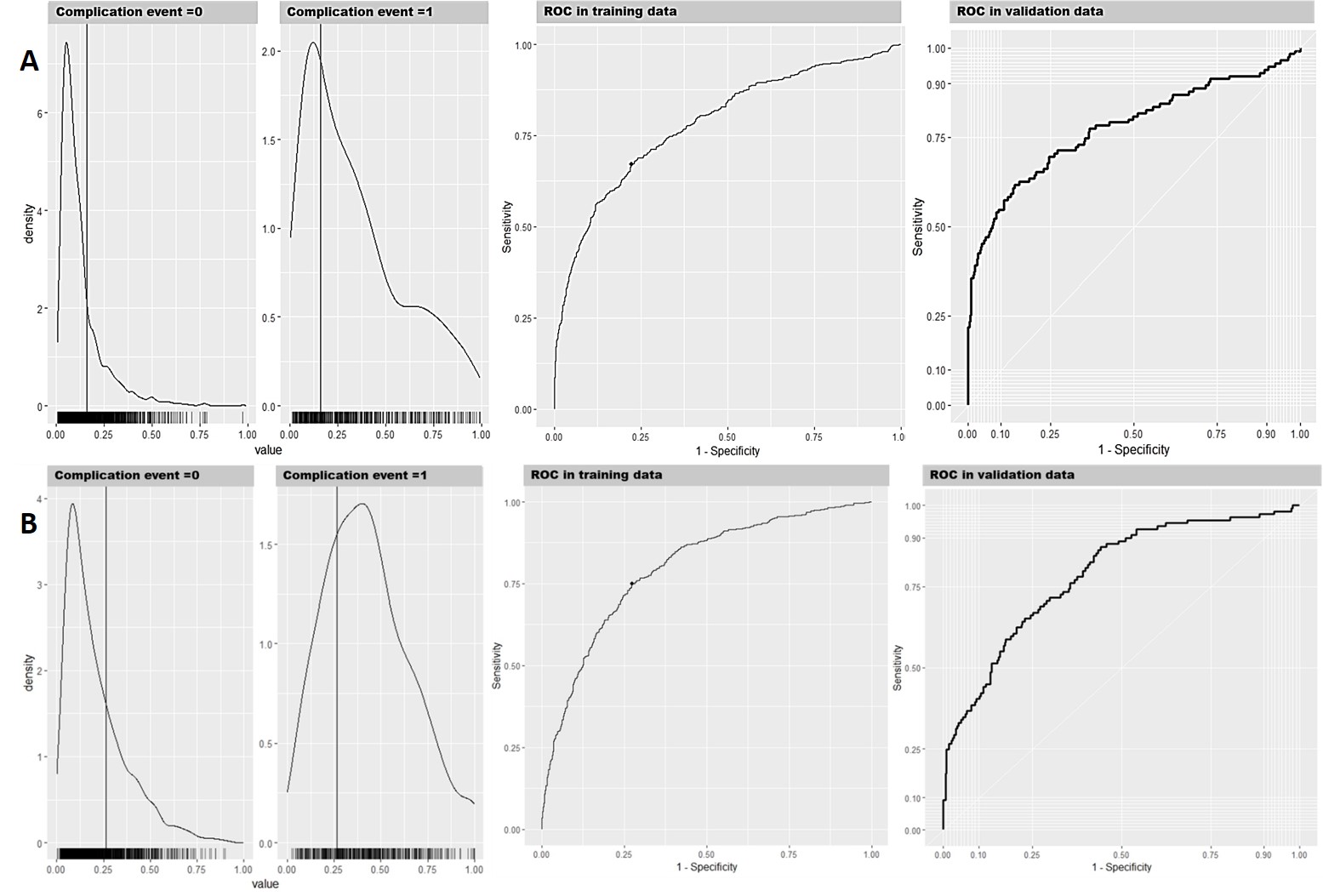

Supplement: Supplementary file 1 — Figure S1: The distribution of the predictor values by adverse events and receiver operating characteristic (ROC) curve for predicting adverse events after blunt chest trauma (A) Model 1: penalized logistic model without hemogram (penalty factor = 8.00) (B) Model 2: penalized logistic model with hemogram (penalty factor = 9.00). [file KJM2-41-e70088-s001.jpg]
